# Clinical phase I/II trial to investigate neoadjuvant intensity-modulated short term radiation therapy (5 × 5 gy) and intraoperative radiation therapy (15 gy) in patients with primarily resectable pancreatic cancer - NEOPANC

**DOI:** 10.1186/1471-2407-12-112

**Published:** 2012-03-23

**Authors:** Falk Roeder, Carmen Timke, Ladan Saleh-Ebrahimi, Lutz Schneider, Thilo Hackert, Werner Hartwig, Annette Kopp-Schneider, Frank W Hensley, Markus W Buechler, Juergen Debus, Peter E Huber, Jens Werner

**Affiliations:** 1 Clinical Cooperation Unit Radiation Oncology, German Cancer Research Center (DKFZ), Heidelberg, Germany; 2 Department of Radiation Oncology, University of Heidelberg, Heidelberg, Germany; 3 Department of Surgery, University of Heidelberg, Heidelberg, Germany; 4 Department of Biostatistics, German Cancer Research Center (DKFZ), Heidelberg, Germany

## Abstract

**Background:**

The current standard treatment, at least in Europe, for patients with primarily resectable tumors, consists of surgery followed by adjuvant chemotherapy. But even in this prognostic favourable group, long term survival is disappointing because of high local and distant failure rates. Postoperative chemoradiation has shown improved local control and overalls survival compared to surgery alone but the value of additional radiation has been questioned in case of adjuvant chemotherapy. However, there remains a strong rationale for the addition of radiation therapy considering the high rates of microscopically incomplete resections after surgery. As postoperative administration of radiation therapy has some general disadvantages, neoadjuvant and intraoperative approaches theoretically offer benefits in terms of dose escalation, reduction of toxicity and patients comfort especially if hypofractionated regimens with highly conformal techniques like intensity-modulated radiation therapy are considered.

**Methods/Design:**

The NEOPANC trial is a prospective, one armed, single center phase I/II study investigating a combination of neoadjuvant short course intensity-modulated radiation therapy (5 × 5 Gy) in combination with surgery and intraoperative radiation therapy (15 Gy), followed by adjuvant chemotherapy according to the german treatment guidelines, in patients with primarily resectable pancreatic cancer. The aim of accrual is 46 patients.

**Discussion:**

The primary objectives of the NEOPANC trial are to evaluate the general feasibility of this approach and the local recurrence rate after one year. Secondary endpoints are progression-free survival, overall survival, acute and late toxicity, postoperative morbidity and mortality and quality of life.

**Trial registration:**

NCT01372735.

## Background

Pancreatic cancer is the fourth leading cause of cancer death in both men and women [1,2]. The only way to cure is complete surgical resection, but only about 15-20% of the patients are amendable to primary surgery at presentation [3]. Even in this prognostic favourable group, five-year survival rates are below 25% [4]. On the one hand, these patients are at high risk for local recurrence, which develops in up to 50% despite aggressive surgical approaches [5,6] and built the rationale for an additive use of radiation therapy. Especially in the US, postoperative chemoradiation has emerged as the standard of care after a survival benefit had been shown compared to surgery alone in the GITSG trial [7]. On the other hand, these patients are also at high risk for distant metastases, which built the rationale for an additive use of systemic therapy. In most European countries, postoperative chemotherapy has emerged as the standard of care because of its survival benefit compared to surgery alone [8,9] and the absence of well-designed randomised trials showing a benefit for adjuvant chemoradiation vs chemotherapy alone. However, the low acceptance of chemoradiation as an adjunct to surgery in Europe, may be caused by some disadvantages because of its postoperative rather than its principle use. Postoperative administration of radiation therapy dose not only cause a delay in dose-intense chemotherapy, but has several limitations in terms of target volume definition as well as in total dose because of the low tolerance of surrounding organs at risk [10].

These limitations have lead to preoperative radiation strategies in several other diseases, for example in rectal cancer [11,12], which offer several advantages, for example an easier target volume definition, less toxicity to adjacent organs at risk because of their displacement by the tumor itself and the possible use of smaller safety margins [13], devitalisation of tumor cells prior to surgery [14], improved response due to better tissue oxygenation [13] and the avoidance of treatment delay due to postoperative complications [15,16]. In pancreatic cancer, such neoadjuvant strategies have mainly been investigated in locally advanced cases because of their potential to down size tumor masses and resulted in secondary resectability in 30-80% of the patients [17], but their principle advantages compared to postoperative approaches have also been confirmed in resectable cases [18]. However, the delay of surgery and chemotherapy by long course radiation therapy schedules seem to be a matter of concern. Hypofractionated short course radiation regimens can overcome this limitation and have shown equal benefit in terms of local control with less acute side effects for example in rectal cancer [12,19,20]. One of the most common short course radiation regimens in rectal cancer uses 5 fractions of 5 Gy each during one week [12]. Using the linear-quadratic model, the biological effect of this regimen corresponds to about 40 Gy in conventional fractionation [21], which is less than the commonly used 50-54 Gy with long term schedules. However, given the high rate of microscopically incomplete resections in pancreatic cancer (up to 75%) [4,22-24], external beam radiation therapy approaches alone seem to be insufficient regardless of their fractionation schedule because of their general dose limitations caused by the low tolerance of the surrounding organs at risk like small bowel and kidneys [25].

To overcome this general limitation, an intraoperative electron boost (IORT) has been introduced successful into the treatment of several malignancies arising in the abdominal cavity. IORT means the application of a high single dose (usually 10 to 20 Gy) under visual control during a surgical intervention exploiting the possibility to displace organs at risk with low radiation tolerance completely from the radiation fields. Considering the increased biological effectiveness of a high single dose [26], total doses which are equivalent to 60-70 Gy in conventional fractionation can be achieved using an IORT boost in combination with moderate doses of external beam radiation therapy [27]. The combination of IORT and EBRT has been shown to improve local control compared to EBRT alone for example in retroperitoneal sarcoma as proven by a randomized controlled trial [28]. In resectable pancreatic cancer patients, a significant improvement in local control and even in overall survival was found with IORT compared to surgery alone in a retrospective analysis by Reni et al. [29], and a recently published pooled analysis of several european IORT centers concluded that combination of IORT with neoadjuvant EBRT is superior to a combination with postoperative EBRT [25]. Therefore the aim of this study is to investigate a neoadjuvant short course EBRT schedule (5 × 5 Gy), followed by surgery with an intraoperative electron boost (15 Gy) in patients with resectable pancreatic cancer, in order to reduce the local recurrence rate. The potential advantage of a neoadjuvant regimen compared to a postoperative schedule consists of a simplified target volume definition, improved sparing of dose to surrounding organs at risk and the devitalisation of tumor cells prior to surgery. The potential advantage of a short course regimen compared to a long course schedule consists of a shorter delay of surgical and dose-intense systemic therapy and a decreased risk of acute radiation side effects and surgical morbidity due the short latency between irradiation and surgery. The combination with an intraoperative boost yields total doses which should be able to control even microscopic residual disease without harm to the adjacent organs at risk. The clinical trial will further be accompanied by the examination of blood and tissue samples before, during and after the radiation treatment using gene expression profiling in order to identify possible predictive factors for treatment response and outcome.

## Methods/Design

### Study design

The purpose of the study is to investigate the value of a neoadjuvant hypofractionated short course radiation therapy schedule followed by immediate surgery and an intraoperative boost to the tumor bed in patients with primarily resectable pancreatic cancer. The trial will be performed as a single-center one-armed phase I/II study.

### Patient selection and inlcusion/exclusion criteria

46 patients fulfilling the inclusion criteria listed below should be enrolled in this trial. Only patients meeting all of the inclusion criteria and missing all of the exclusion criteria (see Table 1) will be considered for admission to the trial.

**Table 1:** Inclusion and Exclusion Criteria

Inclusion criteria	Exclusion criteria
• written informed consent • histologically confirmed, primary pancreatic cancer of the pancreatic head • judged as gross completely resectable • absence of lymph node metastases at the splenic hilum or along the pancreatic tail • no evidence of distant metastases • age > 50 years • Karnofsky performance score ≥ 70% • adequate bone marrow function (neutrophils > 2000/μl, platelets > 100000/μl) • adequate renal function (Creatinine < 1.5 mg/dl) adequate liver function	• missing written informed consent • missing histological conformation of pancreatic cancer • judged as gross incomplete or not resectable • pancreatic cancer located in the pancreatic corpus or tail • recurrent pancreatic cancer • incomplete staging • presence of lymph node metastases along the pancreatic tail or splenic hilum • presence of distant metastases • prior radiation therapy to the upper abdominal region • neoadjuvant chemotherapy or immunotherapy • participation in another clinical interventional study • age ≤ 50 years • other previous or active malignancy (excluding basal cell carcinoma, carcinoma in situ of the cervix) • Karnofsky performance score < 70% • inadequate bone marrow function • inadequate renal or liver function • any other disease or situation, which generally prohibits the use of major surgery or radiation therapy according to the judgement of a surgeon or radiation oncologist • inability to participate in regular follow up • pregnancy, inability or incompliance for adequate contraception • missing ability to give informed consent legal custody

### Trial organization

NEOPANC has been designed by the Departments of Radiation Oncology and Biostatistics at the German Cancer Research Center (DKFZ) in cooperation with the European Pancreas Center and the Departments of General Surgery and Radiation Oncology at the University of Heidelberg. It will be carried out by the Department of Radiation Oncology at the German Cancer Research Center (DKFZ) together with the European Pancreas Center and the Departments of Radiation Oncology and General Surgery at the University of Heidelberg. The trial is an investigator initiated trial.

### Coordination

The trial is coordinated by the Department of Radiation Oncology at the German Cancer Research Center (DKFZ) and the European Pancreas Center at the University of Heidelberg in cooperation with the Departments of General Surgery and Radiation Oncology of the University of Heidelberg. The Department of Radiation Oncology at the German Cancer Research Center (DKFZ) and the European Pancreas Center at the University of Heidelberg are responsible for overall trial management, database management, quality assurance, reporting and for the scientific program of all trial related meetings. The trial has been registered at ClinicalTrials.gov [NCT 01372735].

### Investigators

Patients will be recruited by the European Pancreas Center and the Department of General Surgery at the University of Heidelberg. All investigators are experienced oncologists from the field of radiation oncology or surgery cooperating in this trial.

### Ethical and legal considerations

The study protocol was approved by the independent ethics committee of the medical faculty at the University of Heidelberg and the German Federal Authorities for Radiation Protection (Bundesamt fuer Strahlenschutz). The trial will be carried out by adhering to local legal and regulatory requirements. The study complies with the Declaration of Helsinki 2008, the principles of Good clinical practice (GCP) and the German Federal Data Protection Act. Written informed consent will be obtained from each patient before inclusion into the trial after nature, scope and possible consequences of participation in the trial have been explained by a physician.

### Study objectives and endpoints

The primary objective is to evaluate the local recurrence rate after 1 year. Secondary objectives are general feasibility of this approach, acute and late toxicity, perioperative morbidity and mortality, progression-free survival, overall survival, quality of life and the identification of prognostic biomarkers.

### Pretreatment evaluation

Initial work up consists of clinical examination, laboratory tests including tumor markers, CT or MR-imaging of the abdominal cavity, thoracic CT, assessment of baseline quality of life, evaluation of general ability to receive major surgery and evaluation of resectability.

### Treatment assignment and schedule

**Figure 1:**
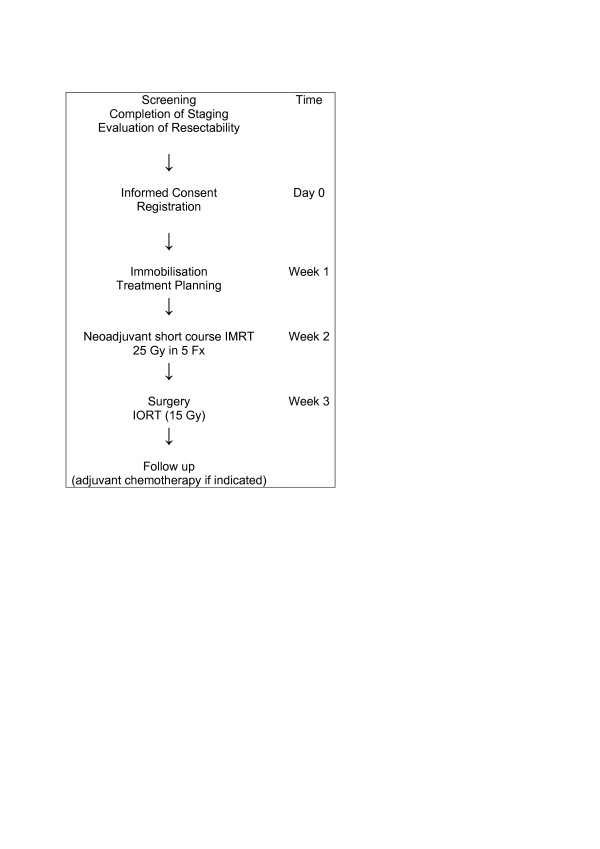
**Flow Chart of the NEOPANC Study**. IMRT: intensity-modulated radiation therapy, IORT: Intraoperative Radiation Therapy, Gy: Gray, Fx: Fractions.

All patients will be assigned to the same treatment regimen in this trial (see Figure 1). Patients who fulfill the inclusion criteria after pretreatment evaluation and gave written informed consent will receive immobilization and treatment planning examinations for neoadjuvant short course radiation therapy within 1 week after registration at the Department of Radiation Oncology, German Cancer Research Center. Neoadjuvant intensity-modulated short course irradiation will start within 1 week after treatment planning and will be carried out in 5 fractions during one week using an image guided intensity-modulated radiation technique up to a total dose of 25 Gy. Surgical resection of the tumor including intraoperative radiation therapy (IORT) with a single dose of 15 Gy will be carried out within 1 week after the end of neoadjuvant irradiation at the department of surgery in cooperation with the department of Radiation Oncology at the University of Heidelberg. After postoperative recovery, all patients will be assigned to regular follow up visits either at the department of Radiation Oncology at the German Cancer research center or at the department of Surgery at the University of Heidelberg. Also all patients will be scheduled to receive postoperative systemic therapy according to the german pancreatic cancer treatment guidelines [30], although systemic treatment is not an inherent part of the study protocol and will be given independently from study participation.

### Neoadjuvant short course radiation therapy

For external beam radiation therapy, patients will be immobilized using an individual vacuum pillow. For treatment planning, contrast enhanced CT as well as MR imaging will be performed for optimal target definition. Organs at risk like small bowel, stomach, liver, kidneys and spinal cord will be contoured. The CTV includes the gross tumor volume and the regional lymph node areas. A safety margin of 1 cm will be added to obtain the PTV. The margin maybe reduced with respect to anatomical borders or organs at risk at the discretion of the investigator. The prescribed dose to the PTV is 25 Gy in 5 fractions with the 95% isodose surrounding the PTV contour. The dose constraint to the spinal cord is 15 Gy in maximum. The median dose to kidneys and liver should be below 7 and 10 Gy, respectively. Only one third of the kidney is allowed to get more than 15 Gy. The minimum and maximum doses inside the PTV should not exceed the range of 95% to 107%. Inverse treatment planning will be done using the planning systems at the Department of Radiation Oncology of the German Cancer Research Center. Treatment will be performed using step-and-shoot intensity modulated radiation therapy with 5 to 9 coplanar 6 MV-beams after stereotactic target point localisation. Daily setup correction will be done using an image-guided approach with an In-Room-CT on rails (Siemens Somatom Emotion, Siemens, Erlangen, Germany) by comparison of the current CT with the planning CT prior to every fraction.

### Surgery and intraoperative radiation therapy

Surgery will be performed within one week after completion of neoadjuvant irradiation at the Department of General Surgery of the University of Heidelberg. A curative resection either as pancreatic head resection or total pancreatectomy is attempted. An additional intraoperative electron boost will be performed using a dedicated linear accelerator inside the operation theatre (Siemens Mevatron, Siemens, Concord, USA). The target volume of the intraoperative boost includes the tumor bed, the celiac and superior mesenteric origins, the mesenteric root, the portal vein and the adjacent paracaval and paraaortic lymph node regions. Therefore an applicator of appropriate size will be placed inside the abdominal cavity and attached to the table by a radiation oncologist in correspondence with the treating surgeon. Uninvolved radiosensitive tissues will be removed from the treatment area or covered by lead shielding. After alignment with the accelerator, the target volume will be irradiated with a single dose of 15 Gy, prescribed to the 90% isodose. The electron energy will be selected according to the tissue depth that has to be covered in order to encompass the target volume with the 90% isodose.

### Follow up

Regular follow up visits will start at discharge of the hospital stay after surgery and will take place every 3 months after surgery for the first 3 years and every 6 months for two further years. They will include clinical examination, CT or MR-imaging of the abdominal cavity, laboratory examinations including Ca 19-9 measurements, assessment of acute/late toxicity and quality of life as well as any other necessary test or examination in case of clinical or biochemical suspicion of local recurrence or distant metastases (at least CT or MR-imaging of lungs and brain and bone scan). All patients will be followed for 5 years or until death or end of study participation due to other reasons.

### Assessment of efficiacy

Local recurrence rate after 1 year is the primary endpoint of the trial. It will be assessed by repeated CT or MR-imaging during regular follow up. In case of a single suspicious locoregional lesion, histological confirmation will be attempted. Otherwise, new lesions with typical radiological signs of a local recurrence in combination with or without a rise of tumormarkers (depending on the tumormarker status before start of treatment) will be counted as local recurrence. In case of missing surgical resection of the tumor after neoadjuvant radiotherapy, a local disease progression according to RECIST criteria will be counted as a local recurrence.

Progression free survival is a secondary endpoint of the study. Progression-free survival will be counted from the first day of radiation therapy until the date of the first event either locally or distantly or death due to any cause. Patients alive without progressive disease at the time of data analysis will be censored at the time of the most recent follow up. In case of lesions suspicious for distant metastases, histological or cytological confirmation is attempted. Otherwise, new lesions with typical radiological signs of distant metastases with or without a rise of tumormarkers (depending on the tumormarker status before start of treatment) will be counted as events. Notably, the sole rise of tumormarkers without any clinical or radiological evidence for a local or distant progression or the sole occurrence of effusions like ascites without a cytological confirmation will not be counted as an event, especially during adjuvant systemic therapy.

Overall survival is a secondary endpoint of the study. The duration of survival is the time interval from the first day of radiation treatment until death of any cause. Patients not reported dead or lost to follow-up will be censored at the date of the last follow-up examination.

### Assessment of toxicity

Acute radiation toxicity will be assessed according to Common Terminology Criteria for Adverse Events Version 3.0 during the time period from the first day of neoadjuvant radiation treatment until 3 months after surgery or until start of adjuvant systemic treatment. Late radiation toxicity will be scored according to RTOG criteria. Toxicity will regularly be evaluated by clinical and laboratory examinations after the first, third and fifth fraction of neoadjuvant radiation therapy. Postoperative morbidity will be assessed at discharge of the hospital stay and 3 months after surgery and late toxicity will be scored during every further regular follow up visit.

### Assessment of quality of life

Measurement of quality of life is one of the secondary objectives of the trial. Two questionnaires are used in this study to assess QoL. EORTC QLQ-C30 is a general measure of quality of life in cancer patients. It incorporates nine multi-item scales: five functional scales (physical, role, cognitive, emotional, social); three symptom scales (fatigue, pain, nausea and vomiting); and a global health scale [31]. Specific symptoms (dyspnea, insomnia, anorexia, constipation, diarrhea, financial impact) are measured as six single items. This instrument has been used in a variety of trials so far and showed a good internal consistency and good re-test reliability [32]. The second questionnaire is a specific pancreatic module (QLQ-PAN26) [33], which has been designed to be used together with the general measure to assess disease-specific symptoms in patients with pancreatic cancer.

### Safety and discontinuation of treatment

Toxicities are classified by type, grade, duration, onset and relationship to radiation treatment. Study participation will be terminated in an individual patient, if the patient suffers from a grade 4 toxicity related to radiation treatment or if the patient withdraws consent to further participation. The trial itself will be aborted if a patient suffers from a grade 5 toxicity related to radiation therapy or if more than 2 of the first 10 patients, more than 4 of the first 20 patients or more than 20% of the further patients suffer from grade 4 toxicities related to radiation therapy. A temporary discontinuation of the study with a consecutive safety analysis will be performed if ≥ 3 of 10 consecutive patients turn out to be not grossly resectable during surgery.

### Statistical considerations and sample size estimation

The primary endpoint of the trial is the local recurrence rate after one year. The study is designed to demonstrate that an additional radiation therapy consisting of neoadjuvant short-term external beam radiation therapy and intraoperative radiation therapy can improve the local recurrence rate after one year. The local recurrence rate after one year in a comparable patient population treated with standard procedures (surgery followed by adjuvant chemotherapy) was estimated to be 30% according to the literature. The sample size calculation was designed on the assumption to detect a decrease of the local recurrence rate after one year by 20% (from 30% to 10%) with a power of 90%. Using the two-sided binomial test with a level of significance of α = 5%, the study requires 40 patients. Assuming a drop-out rate of 15% of patients, who will not be available for the evaluation of local recurrence after one year because of death by distant disease progression or other reasons, the total number of patients recruited for the trial will be 46. Secondary endpoints are all of explorative nature and reported using descriptive methods. Time to event data (progression free survival, overall survival) will be evaluated using the Kaplan-Meier-Method.

### Biomarker

Even in the favourable patients group with primarily resectable pancreatic cancer, long term survival is limited to about 15-20% of the patients due to high rates of local and distant failures. Additional treatments like chemotherapy or radiation therapy could improve the outcome at least in subsets of patients. However, those treatments can be accompanied by substantial side effects leading to reduced quality of life. Therefore it seems important to identify subsets of patients who are more likely to profit from an additional treatment. To identify predictors of outcome, blood and tissue samples of each patient will be examined. Blood samples will be taken before the first day, after the third and the fifth day of neoadjuvant external beam radiation therapy, before surgery and at discharge from the hospital stay after surgery. Tissue samples will be taken during surgery.

## Competing interests

The authors declare that they have no competing interests.

## Authors' contributions

FR planned the study, prepared the study protocol, conducted the correspondence with the legal authorities and prepared the manuscript. PEH planned the study, prepared the parts of the study protocol regarding the accompanying biological program and assisted in preparing the manuscript. JW planned the study, prepared the parts of the study protocol regarding surgical and quality of life aspects and assisted in preparing the manuscript. CT and LSE assisted in preparing the study protocol and the manuscript regarding the (radio)-biological aspects of the study. FWH assisted in preparing the study protocol and the manuscript regarding the aspects of radiation physics in the study. AKS performed the biostatistical parts of the study design, prepared the corresponding parts of the study protocol and assisted in preparing the manuscript. LSC, TH and WH assisted in preparing the study protocol and the manuscript regarding surgical and quality of life aspects. JD and MWB participated in study conception and design. The last two authors contributed equally. All authors read and approved the final manuscript.

## Pre-publication history

The pre-publication history for this paper can be accessed here:

http://www.biomedcentral.com/1471-2407/12/112/prepub
